# Disruption of intestinal oxygen balance in acute colitis alters the gut microbiome

**DOI:** 10.1080/19490976.2024.2361493

**Published:** 2024-07-03

**Authors:** Wenjing Zong, Elliot S. Friedman, Srinivasa Rao Allu, Jenni Firrman, Vincent Tu, Scott G. Daniel, Kyle Bittinger, LinShu Liu, Sergei A. Vinogradov, Gary D. Wu

**Affiliations:** a Division of Gastroenterology, Hepatology, and Nutrition, The Children’s Hospital of Philadelphia, Philadelphia, PA ,USA; b Department of Gastroenterology & Hepatology, Perelman School of Medicine, University of Pennsylvania, Philadelphia, PA, USA; c Department of Chemistry, School of Arts and Sciences, University of Pennsylvania, Philadelphia, PA, USA; d Department of Biochemistry and Biophysics, Perelman School of Medicine, University of Pennsylvania, Philadelphia, PA, USA; e Dairy and Functional Foods Research Unit, Eastern Regional Research Center, Agricultural Research Service, US Department of Agriculture, Wyndmoor, PA, USA

**Keywords:** Intestine, colitis, oxygen, glycoside hydrolase, phosphorescence, microbiota

## Abstract

The juxtaposition of well-oxygenated intestinal colonic tissue with an anerobic luminal environment supports a fundamentally important relationship that is altered in the setting of intestinal injury, a process likely to be relevant to diseases such as inflammatory bowel disease. Herein, using two-color phosphorometry to non-invasively quantify both intestinal tissue and luminal oxygenation in real time, we show that intestinal injury induced by DSS colitis reduces intestinal tissue oxygenation in a spatially defined manner and increases the flux of oxygen from the tissue into the gut lumen. By characterizing the composition of the microbiome in both DSS colitis-affected gut and in a bioreactor containing a stable human fecal community exposed to microaerobic conditions, we provide evidence that the increased flux of oxygen into the gut lumen augments glycan degrading bacterial taxa rich in glycoside hydrolases which are known to inhabit gut mucosal surface. Continued disruption of the intestinal mucus barrier through such a mechanism may play a role in the perpetuation of the intestinal inflammatory process.

## Introduction

The interaction between the mammalian host and its microbiota is complex and multifactorial, particularly in the distal gut where well-oxygenated intestinal tissue is directly adjacent to the anaerobic environment of the gut lumen. The gut lumen is deeply anaerobic largely due to the consumption of oxygen by the gut microbiota, although depletion of oxygen due to chemical reactions (e.g., oxidation of organic substrates) may also play a role.^1^ Undoubtedly, there exists an oxygen gradient along which the diffusion of oxygen occurs from the intestinal mucosa into the lumen of the gut where it is consumed. At the interface, the mucosal surface of the distal gut is, therefore, likely to be a microaerobic environment. Histological stains of intestinal tissue which are consistent with tissue hypoxia at the surface epithelium,^2^ immunolocalization of bacterial response to oxygen at the mucosal surface,^3^ and the enrichment of facultative anaerobic bacterial of mucosally adherent microbiota relative to fecal material in the distal gut lumen,^1,4,5^ provide indirect evidence for this notion. Even the intestinal epithelium has been proposed to be a sink for oxygen, thereby reducing the flow of oxygen from intestinal tissue into the gut lumen, due to its ability to utilize gut microbiota generated short chain fatty acids as metabolic fuels for oxidative metabolism.^6^

The homeostatic balance of oxygen between host intestinal tissue and the gut microbiota is likely disrupted during intestinal inflammation, although the actual alterations have not been well characterized. From the host perspective, a decrease in oxygenation of the surface epithelium due to a disruption of the intestinal microvasculature has been suggested to exacerbate the hypoxemic nature of this epithelial compartment.^2^ Alternatively, alteration of gut microbiota composition during intestinal inflammation could reduce short chain fatty acid production, reduce oxygen consumption by the intestinal epithelium, and lead to an increase in oxygenation of the intestinal lumen.^6,7^ Does intestinal inflammation increase the delivery of oxygen into the gut lumen, or does it lead to a decrease? The increase in the abundance of facultative anaerobes belonging to the *Enterobacteriaceae* clade associated with intestinal inflammation in patients with IBD as well as in animal models,^6,8,9^ commonly referred to as “dysbiosis”, would suggest that oxygen delivery might be increased. However, it is well established that intestinal inflammation leads to the production of molecules that can serve as alternative electron acceptors, thus promoting anaerobic respiration leading to enhanced growth of *Enterobacteriaceae*^6^ - a biological process that would only occur if the gut lumen remained anaerobic in the setting of intestinal inflammation. Quantification of intestinal luminal oxygenation in the setting of inflammation would help to place these seemingly opposing mechanisms within the appropriate physiological context.

Previously, we have used the phosphorescence quenching method to quantify oxygen levels in intestinal tissue and the lumen of the gut in real time,^1,4^ but not simultaneously in the same animal. These studies have shown that the distal gut lumen is anaerobic due to both the gut microbiota and oxygen-consuming chemical reactions (spontaneous oxidation of organic substrates), and that alterations of host oxygenation can transiently influence luminal oxygen levels. Herein, through the use of the recently developed two-color phosphorometry,^10^ which was specifically adapted in this work to measure oxygen levels in both intestinal tissue and the gut lumen simultaneously, we determined the relationship between intestinal tissue and gut luminal oxygenation in the setting of intestinal inflammation in the dextran sodium sulfate (DSS) model of colitis. Both the average tissue partial oxygen pressure (pO_2_) and the magnitude of its response to changes in the oxygen content in the inhaled gas mixture were reduced in colitis consistent with disruption of the intestinal microvasculature. Although oxygen levels in the intestinal lumen remained low during intestinal inflammation, hyperoxygenation of the host lead to a statistically significant increase in luminal pO_2_, which may be an indication of alterations in the dynamics of host–microbiota interactions in the setting of intestinal inflammation. Indeed, shotgun metagenomic sequencing of the gut microbiota revealed that DSS colitis led to significant alterations primarily in the *Bacteroidetes* and *Verrucomicrobia* phyla. Similar alterations were seen *in vitro* using a human colonic microbial community exposed to a microaerobic environment in an intermittently pulsed flow cultivar apparatus. This suggests existence of a previously uncharacterized relationship between oxygen and bacterial glycan degradation relevant to intestinal mucus biology at the mucosal surface. In total, our results demonstrate that intestinal inflammation alters host tissue oxygenation that, in turn, can influence the composition of the luminal microbiota.

## Results

### Alternations in tissue and luminal oxygenation of the mouse cecum induced by inflammation

**Figure 1.**
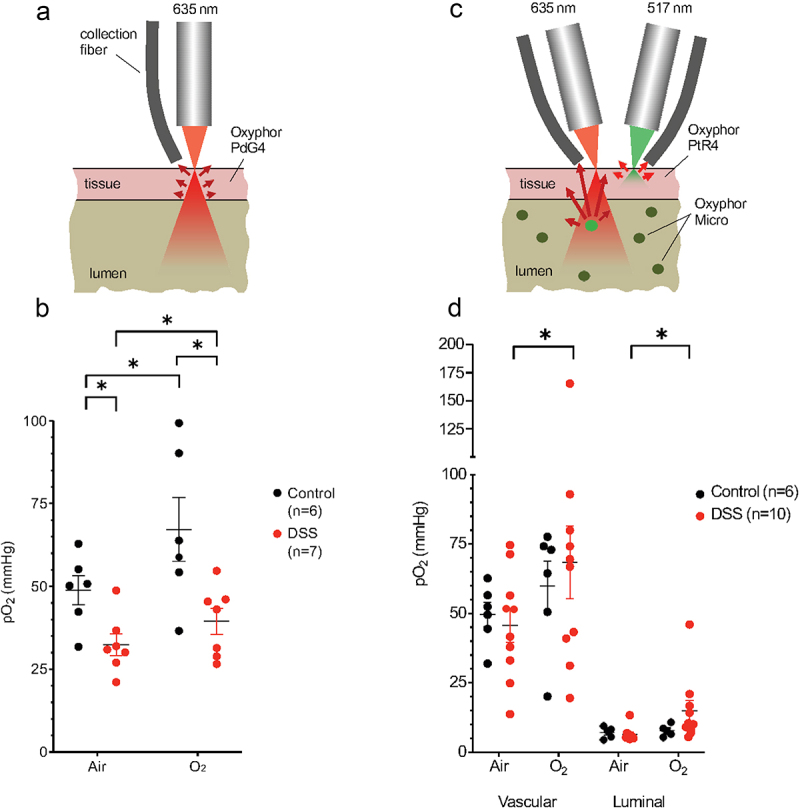
Effects of DSS colitis on deep vascular, shallow vascular, and luminal oxygen levels quantified by phosphorescence quenching oximetry. (a) Schematic diagram of deep tissue oxygen sampling using Oxyphor PdG4 probe throughout the entire thickness of the cecum. (b) Vascular oxygen levels in control and DSS-treated mice under both ambient air and 100% oxygen inspiration as measured using Oxyphor PdG4. (c) New two-color phosphorometry allows for simultaneous quantification of shallow vascular (Oxyphor PtR4) and intraluminal oxygen levels (Oxyphor Micro). (d) Shallow vascular and luminal oxygen levels in control and DSS treated mice breathing ambient air and 100% O_2_. *FDR corrected p < .05 by unpaired or paired Student’s t-tests with two-stage step-up correction for multiple comparisons.

When injected systemically in healthy tissue, dendritic oxygen probes, including Oxyphors PdG4 and PtR4, remain confined to the vasculature for many minutes/hours following the injection. In tissues with disrupted vasculature the probes partially leak out into the interstitial space. Excitation light at ~635 nm, which is used to excite PdG4, can penetrate through many millimeters of tissue.^11^ Therefore, measurements with PdG4 injected in the blood represent average intestinal tissue pO_2_, mostly in the vascular compartment, including layers immediately adjacent to the lumen (Figure 1a). In the case of PtR4, excitation is carried out using 517 nm light, which is able to diffuse only through the superficial tissue layers, sampling pO_2_ only in the upper ~ 50 μm of the tissue,^10^ and hence excluding the deeper layers adjacent to the lumen (Figure 1c).

First, we carried out measurements using PdG4 in the blood, as described previously.^1,4^ The average tissue oxygenation in the cecum increased significantly upon switching respiration from ambient air to 100% O_2_ (Figure 1b), congruent with previous observations.^1,4^ We then made the same measurements in the setting of intestinal inflammation using the dextran sodium sulfate (DSS) model of colitis, which is typically manifested by a reduced weight, shortening of the cecum and colon, histologic evidence of acute inflammation, and loss of the cecal epithelium (Supplemental Figure S1). In this case, we also observed a significant increase in tissue oxygenation in the cecum upon with respiration of 100% O_2_ (Figure 1b). On average, tissue oxygenation was significantly lower in mice with DSS colitis compared to healthy controls when both were breathing ambient air (Figure 1b). These results are consistent with disruption of the intestinal microvasculature in the setting of colitis,^12,13^ although the relative change in tissue oxygenation appears to remain intact upon switching the inhaled gas to 100% O_2_ (Figure 1b).

The effect of intestinal inflammation on intraluminal oxygen concentrations has not been previously described. Since the level of tissue damage induced by DSS colitis can be variable between mice, in this work we ventured to perform simultaneous quantification of oxygen in both cecal tissue and the lumen in the same animals. For that, two different phosphorescent probes with non-overlapping optical spectra were used: Oxyphor PtR4^10^ for tissue and Oxyphor Micro^1,4^ for luminal oxygen measurements (Figure 1c). Unlike soluble Oxyphors (PtR4 or PdG4), Oxyphor Micro comprises solid particles, which were dispersed in the lumen. The phosphorescent chromophore in Oxyphor Micro is the same as that in PdG4, having the excitation maximum at 635 nm. Therefore, measurements with Oxyphor Micro in the lumen sampled through the entire luminal volume, including the intestinal mucosa (Figure 1c).^4^

In healthy controls, the superficial tissue oxygen levels, assessed with PtR4, were similar to those measured (in separate experiments) with PdG4 (Figure 1b,d). However, compared to the healthy controls, there was no significant reduction in the superficial tissue pO_2_ levels in mice with DSS (Figure 1d), compared to a significant reduction observed using PdG4 (Figure 1b). This result is consistent with the notion that DSS colitis leads to the alterations in tissue oxygenation in a depth-dependent manner, i.e., the damaged portion of the vasculature is located further away from the exposed tissue surface, closer to the mucosal interface, hence it is reachable with deeper penetrating (red) 635 nm light, but not with (green) 517 nm light. This observation is consistent with independent measurements of tissue oxygen distributions throughout the colon performed by nitroimidazole staining.^2^

Luminal oxygen measurements using Oxyphor Micro revealed overall very low oxygen levels in both control mice and mice with DSS colitis (Figure 1d). Nevertheless, in the setting of DSS colitis, luminal oxygenation increased upon switching from ambient air to 100% oxygen (Figure 1d). This finding suggests that alterations in the luminal environment of the gut, such as increased water content, may lead to an increase in oxygen diffusion to the lumen in the setting of colitis.^1^

### DSS colitis-induced alterations to microbiome composition and the metagenome are consistent with enhanced glycan metabolism

**Figure 2.**
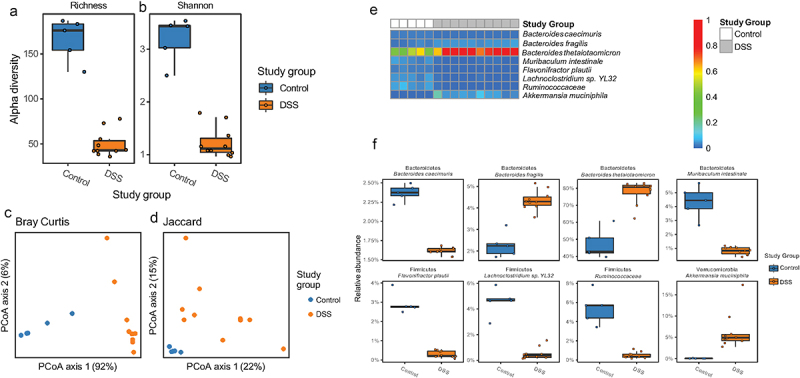
Effect of DSS colitis on the composition of the cecal microbiome. Alpha diversity assessed by (a) richness and (b) Shannon Index (p < .05 for both, two-tailed unpaired Student’s t-tests). Beta diversity assessed by (c) Bray-Curtis and (d) Jaccard distances (p = .01 for both, PERMANOVA). (e) Heatmap showing relative abundance of bacterial species in the cecum of control and DSS treated mice. (f) Differentially abundant taxa between the control and DSS groups (FDR <0.05, mixed effects modeling).

Shallow shotgun metagenomic sequencing of cecal contents showed that DSS colitis significantly decreased microbiome alpha diversity (Figure 2a,b) and altered beta-diversity (Figure 2c,d). *Bacteroides thetaiotamicron, Bacteroides fragilis*, and *Akkermansia muciniphila* were increased in mice with DSS colitis while *Bacteroides caecimuris, Muribaculum intestinale, Flavonifractor plautii, Lachnoclostridium sp*. YL13, and *Ruminococcaceae* were higher in healthy control mice (Figure 2e,f). In the feces of these mice, there were similar but non-significant trends in alpha- and beta-diversity (Supplemental Figures S2a-d) as well as the relative abundances of *B. thetaiotamicron, B. fragilis, A. muciniphila* (increase in DSS group), and *M. intestinale* (decrease in DSS group) (Supplemental Figures S2e,f).

**Figure 3.**
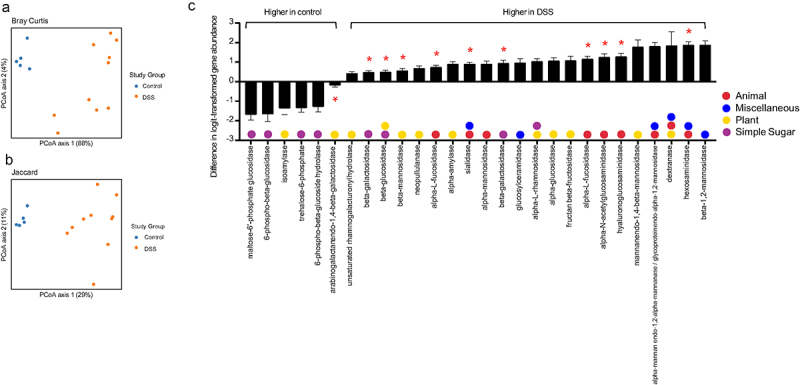
Effect of DSS colitis on the metagenome of the cecal microbiome. PCA plots showing (a) Bray-Curtis and (b) Jaccard distances for the metagenomes of control and DSS-treated mice. (c) Fold change in 28 glycoside hydrolase genes with nonzero counts in at least 90% of samples that were significantly altered with DSS colitis (FDR <0.10). Positive values indicated an increased abundance in the DSS group. Red asterisks indicate genes that are involved in mucin degradation. Glycoside hydrolase genes with multiple colors can act on multiple categories of glycans.

In addition to taxonomic composition, DSS colitis significantly altered the metagenome of the cecal microbiome (Figure 3a,b). Given the alterations in the epithelium and mucus layer in the setting of colitis,^14,15^ as well as the well-known roles of *Bacteroides spp*. and *A. muciniphila* in carbohydrate metabolism,^16^ we chose to focus on glycoside hydrolase genes – which encode enzymes that hydrolyze glycosidic bonds in complex carbohydrates. There were 55 glycoside hydrolase (GH) genes that had nonzero counts in at least 90% of samples; including 28 that were statistically significant on gene abundance between control and DSS colitis following correction for multiple comparisons (Figure 3c, FDR < 0.1). We next categorized these glycoside hydrolase genes as either animal, plant, simple sugar, or miscellaneous based on the carbohydrates they degrade. Of the six GHs that were significantly decreased in the setting of DSS colitis, four are involved in simple sugar metabolism and two are involved in plant glycan metabolism. Of the 22 GHs that were significantly increased in the setting of DSS colitis, nine are involved in animal glycan metabolism, 10 are involved in plant glycan metabolism, four are involved in simple sugar metabolism, and six are involved in miscellaneous glycan metabolism. Specifically, 11 GHs known to be involved in the degradation of mucin^17^ were significantly altered, ten of which increased with DSS colitis (red asterisks, Figure 3c). These changes in GH abundance, along with the alterations in specific microbial taxa known to be involved in complex carbohydrate degradation (i.e., *A. muciniphila*, *Bacteroides spp*.),^16,18^ suggest a fundamental shift in glycan metabolism in the gut during DSS colitis that corresponds with changes in oxygen dynamics at the mucosal surface.

### An in vitro human colonic community exposed to microaerobic oxygen levels recapitulates the taxonomic and metagenomic alterations observed in mouse DSS-induced colitis

We provide evidence that DSS colitis may introduce oxygen into the gut lumen where it is associated with both taxonomic and metagenomic functional alterations in the cecal gut microbiota consistent with altered glycan metabolism (Figures 1–3). Such alterations might also be due to mechanisms associated with host physiology including immune response^19,20^ and/or the production of small molecules that may facilitate anaerobic respiration.^6^ To determine the degree to which a single environmental factor – oxygen delivery – could reproduce the effects on the gut microbiome observed with DSS colitis in the absence of host-factors, we characterized the effect of oxygen on the human gut microbiome *in vitro*. A stable colonic human bacterial community was established in a pulse-fed cultivar under anaerobic conditions, and following a two week stabilization period, oxygen was continuously infused into the community for an additional two weeks (40 mmHg O_2_, approximating mixed venous tissue oxygenation; Figure 1 and Albenberg et al.^4^). However, dissolved oxygen measurements remained at 0% indicating that the community was maintained under anaerobic conditions due to the rapid consumption of oxygen by facultatively aerobic organisms – a phenomena consistent with our observations of luminal oxygen levels in DSS mice under ambient air inhalation (Figure 1d).

**Figure 4.**
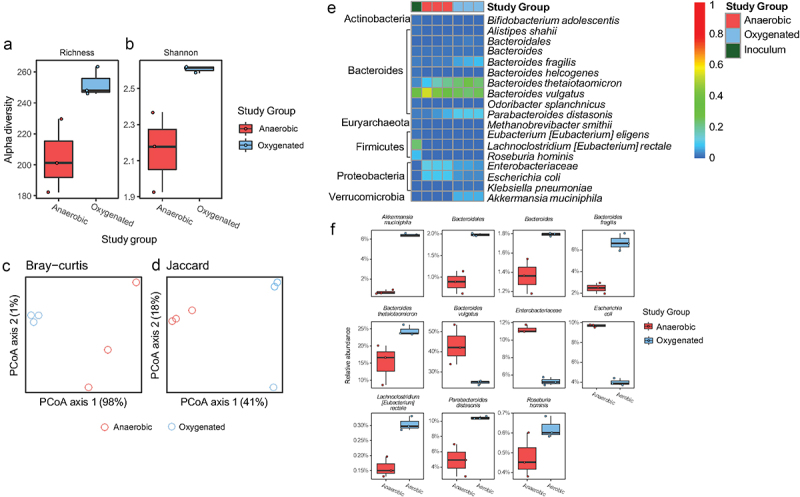
Compositional alteration of a human gut microbiome community in response to oxygenation in vitro. Alpha diversity assessed by (a) richness and (b) Shannon diversity (p < .05 for both, two-tailed Student’s t-tests). PCA plots of beta diversity shown as (c) Bray-Curtis and (d) Jaccard distances (p = .1 for both, PERMANOVA). (e) Heatmap showing relative abundance of genera showing alterations in the microbial community under anaerobic and oxygenated conditions. (f) Differentially abundant taxa between anaerobic and oxygenated conditions (FDR <0.05, linear models of logistic transformed abundances).

In this *in vitro* model, the introduction of oxygen significantly increased alpha diversity (Figure 4a,b). There was also a nonsignificant effect on beta-diversity as visualized by Bray Curtis and Jaccard distances (Figure 4c,d). *A. muciniphila, Bacteroidales spp., Bacteroides spp., B. fragilis, B. thetaiotamicron*, *Eubacterium rectale, Parabacteroides distanosis*, and *Roseburia hominis* were increased under oxygenated conditions while *Bacteroides vulgatus*, *Enterobacteriaceae spp.*, and *Escherichia coli* were decreased under oxygenated conditions (Figure 4e,f). Interestingly, the three bacterial species that were significantly increased in the setting of DSS colitis, *B. thetaiotamicron*, *B. fragilis*, and *A. muciniphila*, also increased significantly upon exposure of the human colonic community to oxygen in our cultivar study (Figures 2f and 4f).

**Figure 5.**
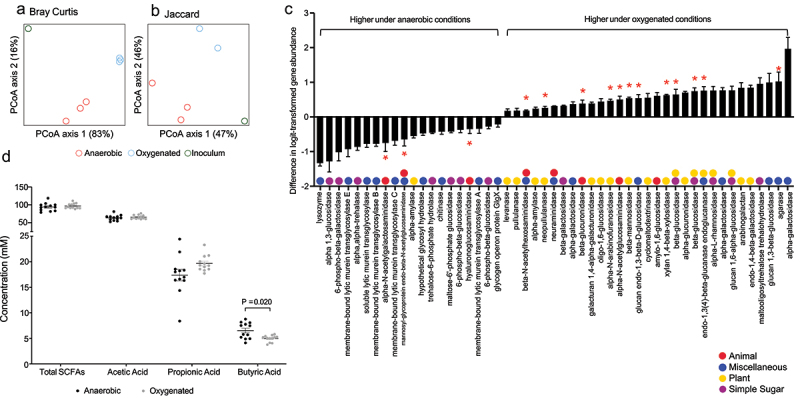
Alteration of a human gut microbiome metagenome and metabolome in response to oxygenation in vitro. PCA plots showing (a) Bray-Curtis and (b) Jaccard distances for the metagenomes under anaerobic and oxygenated conditions. (b) Fold change in 51 glycoside hydrolase genes with nonzero counts in at least 90% of samples that were significantly altered under oxygenated conditions (FDR <0.10). Positive values indicated an increased abundance in under oxygenated conditions. Glycoside hydrolase genes with multiple colors can act on multiple categories of glycans. *Glycoside hydrolases involved in mucin degradation. (d) Short chain fatty acid levels in reactor effluent under anaerobic and oxygenated conditions.

These oxygen-induced taxonomic alterations corresponded with changes in the metagenome of the microbiome (Figure 5a,b). We again analyzed GH gene abundance, and found that, of 66 GHs with nonzero counts in at least 90% of samples, 51 were significantly different between anaerobic and oxygenated conditions (Figure 5c). We categorized these GHs based on their activity and found a similar pattern to the results in mice. Specifically, there was a decrease in seven simple sugar GHs, 10 miscellaneous GHs, three animal GHs, and one plant GH. Meanwhile, there was an increase in 17 plant GHs, five animal GHs, nine miscellaneous GHs, and six simple sugar GHs. Sixteen GHs specifically involved in mucin degradation,^17^ were differentially abundant with 13 of them were increased under oxygenated conditions (red asterisks, Figure 5c). These results in an *in vitro* system, which contains mucin, were similar to those found in mice with DSS colitis. In concert, these findings support the notion that a disruption of the microvasculature resulting in increased oxygen delivery to the lumen during inflammation is, at least in part, responsible for alterations in microbial community structure consistent with an increase in mucin degradation. Finally, we quantified levels of short-chain fatty acids, the end products of bacterial carbohydrate fermentation, under both anaerobic and oxygenated conditions. This revealed decreases in butyric acid, an important source of energy for the colonic epithelium^21,22^ under oxygenated conditions (Figure 5d). Taken together, our findings from *in vivo* animal studies and *in vitro* microbial culture studies suggest that altered mucosal oxygen balance during intestinal inflammation alters the composition and function of the gut microbiome in a manner that may perpetuate intestinal barrier dysfunction through the bacterial degradation of mucus and reducing a short chain fatty acid important in maintenance of colonic epithelial integrity.

## Discussion

The juxtaposition of oxygenated colonic tissue and the anaerobic environment of the gut lumen suggests that there is an oxygen gradient, whereby the surface epithelium of the colon may be relatively hypoxic, and that the adjacent mucus environment may be a microaerobic environment. However, the impact of intestinal inflammation on this gradient has been the topic of much speculation, where some models suggest that there might be an increase in the flux of oxygen into the gut lumen.^6,7^ Indeed, an increase in luminal oxygenation might be one explanation for the increased representation of facultatively anaerobic taxa, such as *Enterobacteriaceae*, in the setting of intestinal inflammation. Alternatively, disruption of the microvascular network within the intestinal tissue with subsequent vascular progenitor hyperproliferation^23–25^ may also influence both tissue oxygenation and release of oxygen into the gut lumen. Herein, using a combination of noninvasive oxygen quantification *in vivo* combined with an assessment of bacterial community dynamics using an *in vitro* bacterial cultivar system, we provide evidence that oxygen flux into the intestinal lumen induced by acute intestinal injury can independently induce a compositional change in the microbiome typified by a signature for enhanced glycan degradation focused on mucus.

We have previously characterized a noninvasive modality to quantify oxygen levels either in intestinal tissue or in the gut lumen in real time using phosphorescence quenching oximetry.^1,4^ In addition to providing a static measurement, this technology allowed us to determine the changes in both tissue and intestinal luminal oxygenation caused by an increase in the inspired oxygen from ambient air to 100% oxygen. The cecum was chosen in this study for practical reasons. First, it is an easily accessible portion of the colon to position the laser used in this study as opposed to other parts of the colon. We wanted to preserve the vasculature and native function of the organ without excessive dissection and manipulation to cause artificial changes in blood flow and subsequent oxygenation. Secondly, the luminal probe was mixed in with luminal contents, which are maximally concentrated in the cecum to create the maximal intensity of signals. Since this is the first time we are using this technology in a disease-model, ability to optimize the conditions for accurate detection was important. The application of this technology to a model of acute intestinal induced by DSS revealed a number of important physiologically relevant processes. Colitis has been associated with an increase in vascularization of the intestinal mucosa^12,13^ suggesting that tissue oxygenation might increase with intestinal injury. Transgenic mice that overexpress vascular endothelial growth factor A (VGEF-A) were shown to have increased angiogenesis, elevated vascular permeability, and greater leukocyte adherence compared with wild type mice leading to a worsening DSS colitis.^26^ These changes to the microvasculature are associated with dilation and increased permeability,^25^ which could result in increased oxygen delivery to the lumen. On the other hand, immunohistochemical analysis of intestinal tissues suggest that colitis leads to intestinal epithelial hypoxia.^2^ By quantifying static levels of tissue oxygenation we show that intestinal injury reduces intestinal tissue oxygenation in and near the mucosa, where vascular disruption has been previously described,^12,13^ but superficial tissue layers, which are more remote from the damaged tissue, remain relatively normoxic. However, responsiveness of tissue oxygenation to inspiration of 100% oxygen remains intact.

Via the simultaneous quantification of both tissue and luminal oxygenation in the cecum of mice, we provide evidence that luminal oxygen levels remain very low in the setting of DSS colitis. This is consistent with the rapid consumption of oxygen by facultative anaerobes known to be enriched at the intestinal mucosal surface.^1,4,5,27^ This finding is consistent with observations in an *in vitro* bacterial cultivar system, where anaerobic conditions are maintained despite the diffusion of physiological oxygen levels (5% or 40 torr) into a human colonic microbiota. Nevertheless, the greater increase in luminal oxygenation induced by the inspiration of 100% oxygen observed in mice with DSS colitis provides evidence that intestinal injury increases oxygen delivery into the gut lumen. The mechanism by which DSS colitis leads to this increase remains to be characterized, but it may include the loss of intestinal epithelial consumption of oxygen with a reduction in oxidative phosphorylation due to a reduction in epithelial mitochondrial function^22^ combined with higher liquid content in the in the cecal feces that would facilitate more rapid diffusion of oxygen.^1^

The characterization of the gut microbiome in the cecum and fecal pellets by shotgun metagenomic sequencing allowed us to determine the effect of colitis on both taxonomy and gene abundance. Rather than observing an increase in the relative proportion of Proteobacteria such as *Enterobacteriaceae* associated with human intestinal inflammatory processes such as IBD and *Clostridioides difficile* colitis^28^ we observed a very robust increase in glycan-degrading taxa such as *Bacteroides*, especially *B. thetaiotaomicron* (approximately 80% of the taxa in DSS colitis), and *A. muciniphila*. Although this may be, in part, to the due to the source of the mice known to be deficient in *Enterobacteriaceae*^29^ it is interesting that our *in vitro* cultivar experiment, where the inoculum contained *Enterobacteriaceae*, showed a decrease in abundance under microaerobic conditions. Such observations suggest that, within the context of a complex community, the increased diffusion of oxygen alone is not sufficient to augment the expansion of facultative anaerobic bacteria such as *Enterobacteriaceae* relative to more obligately anaerobic species. One possible explanation for this response is the relative inability of *Enterobacteriaceae* to utilize complex glycans as a carbon source due the paucity of the appropriate glycoside hydrolases.^10^ Since the composition of carbohydrates in the microbial culture media used in our cultivar study contained primarily complex carbohydrates with low levels of simple carbohydrates (glucose), the robust expansion of *Enterobacteriaceae* such as *E. coli* and *Klebsiella pneumoniae* may not be possible even in the presence of oxygen. Indeed, we have shown that dietary fiber constrains the growth of Proteobacteria spp. upon the reconstitution of the gut microbiota in humans.^30^

The mucin and the complex glycan rich environment of both the cecum of mice and in the culture medium of our cultivar study would favorably promote the growth of glycan-degrading species such as *A. muciniphila*, a mucin specialist, and *B. thetaiotamicron*, a mucin generalist capable of growth on several other polysaccharides.^16^ Since the intestinal mucus environment is believed to be a microaerobic environment, it is perhaps not surprising that such bacterial species would show enhanced growth with the introduction of low levels of oxygen into the environment. Although initially classified as a strict anaerobe, *A. muciniphila* has been shown to not only tolerate but actually show enhanced growth in low levels of oxygen.^31^ *A. muciniphila* responds to the presence of oxygen through two separate mechanisms.^31–33^ First, *A. muciniphila* carries several genes that function to detoxify oxygen, and exposure to oxygen upregulates the oxygen stress response proteins superoxide dismutase, hydroperoxidase, and rubrerythrin.^31,34^ Second, *A. muciniphila* can reduce oxygen through the cytochrome *bd* complex at a capacity of ~ 2.26 ± 0.99 mU mg^−1^ total protein, most likely by using iron-containing heme as cofactors for the reaction.^31^ Similarly, consistent with the increase in abundance to oxygen exposure both *in vitro* and *in vivo*, *B. fragilis* contains the necessary pathways to reduce low levels of oxygen^35^ and *B. thetaiotamicron* has been found to contain a number of enzymes capable of protecting it from reactive oxygen species (ROS) that arise upon exposure to oxygen, such as rubrerythrin.^36^

These observations provide mechanistic insight into previous reports also showing a similar induction of *A. muciniphila* and/or *Bacteroides spp*. with DSS colitis.^34,37–40^ Together, *B. thetaiotaomicron*, *B. fragilis*, and *A. muciniphila* comprised approximately 95% of the taxa in the cecum associated with DSS colitis in our study. The enrichment of numerous glycoside hydrolases, including ten involved in mucin degradation, in the microbiome of mice with DSS colitis is likely a reflection of the ability of these species to inhabit intestinal mucus and where complex glycans are an abundant energy source.^16^ Although the mechanism by which glycan degrading bacteria are enriched in DSS colitis remains to be determined, the ability to reproduce the same induction of both *Bacteroides spp*. as well as *A. muciniphila* in addition to numerous mucus degrading glycoside hydrolases upon the infusion of microaerobic levels of oxygen into a stable human colonic community maintained in a pulse-fed cultivar, suggests that intestinal oxygen levels may play a role. This would certainly be congruent with the enhanced delivery of oxygen into the gut lumen in the setting of DSS colitis that we observed using oxygen phosphorescence quenching.

In total, our results demonstrate that both host intestinal tissue and gut luminal oxygenation are both altered in the setting of colonic injury. Although tissue oxygenation is reduced, there is an increase of oxygen delivery into the gut lumen where it is likely that the consumption by the gut microbiota helps to maintain an anaerobic environment. Nevertheless, the enhanced delivery of oxygen into a complex intestinal bacterial community promotes the enrichment of glycoside hydrolase rich bacterial species capable of mucus degradation which may play a role in the reduction of the mucus barrier function described in patients with IBD thereby augmenting the intestinal inflammatory process.^16,41^

## Materials and methods

### Animal studies

C57BL/6 female mice between 8–12 weeks old were purchased from Jackson Laboratory and housed at the UPenn research animal facility where they were fed a standard chow diet (LabDiet 5001, Land O’Lakes, Inc). For DSS-induced colitis, mice drank vivarium water supplemented with 3.5% DSS (DSS, m.w. 36000–50,000) for 7 days and then returned to normal water on day 7 in preparation for experiments the subsequent day. Mice were examined daily for body weight, water and food intake, activity, fur appearance, and diarrhea, and were scored using the DSS Disease Activity Index (DAI).^42^ All animal use and handling were performed in compliance with National Institute of Health guidelines and with approval of the University of Pennsylvania Institute of Animal Care and Use Committees.

### Histopathology

Cecum and colon samples were fixed in 4% paraformaldehyde solution, embedded in paraffin, sectioned longitudinally, and stained with hematoxylin and eosin. Sections were evaluated and scored for inflammation.

### Oxygen measurements by phosphorescence quenching

In this work we performed two different kinds of oxygen measurement experiments in mice. First, pO_2_ levels in tissue only were measured using a soluble probe Oxyphor PdG4^11^ injected systemically, as described previously.^4^ Secondly, we performed simultaneous measurements of intestinal tissue pO_2_ and luminal pO_2_ by two-color phosphorometry using a combination of two probes, Oxyphor PtR4 (tissue pO_2_) and Oxyphor Micro (luminal pO_2_). The methodology for concurrent oxygen measurements using two probes with non-overlapping optical spectra has been described previously.^10^ Measurements were performed using a time-domain phosphorometer equipped with two laser diodes for excitation (517 nm and 635 nm) and two avalanche phorodiodes (APDs) for detection, as in the previous studies.^10^ The probe for intestinal tissue oxygen measurements, Oxyphor PtR4, has been described previously.^10,43^ The luminal probe was Oxyphor Micro, which comprises solid particles of PMMA doped with Pd tetraaryltetrabenzoporphyrin (PdTBP),^1,4^ whose spectroscopic properties are identical to those of the soluble probe Oxyphor PdG4. In brief, PMMA (Sigma-Aldrich, 17 g) was dissolved in dichloromethane (60 mL), and PdTBP-AG^2^OBu^12^ was added to the resulting solution to the final concentration of ~100 μM. Dichloromethane was removed under vacuum, and the resulting solid was dried in vacuum. The solid PdTBP-containing PMMA pieces were grinded, and particles > 50 μm in diameter were collected by passing the resulting powder through a sieve. The large diameter of the particles (50–100 μm) ensured that the overwhelming majority of the oxygen-sensing species (PtTBP molecules) were located not on the particles’ surfaces, and hence the diffusion of oxygen to these centers was defined solely by the properties of the polymer matrix and not affected by the contacts of the particles with the contents of the gut lumen. It is due to this property that the calibration of the probe in the solid polymer particles *in vitro* could be used to quantify oxygenation of the medium with unknown oxygen diffusivity (gut lumen contents). The luminal PdTBP-PMMA probe was calibrated as described previously.^1,4^

The luminal probe was gavaged into mice through gavage needles at least 5 h prior to the experiment. Oxyphor R4 was injected via the tail vein 5 min prior to oxygen measurements. Anesthesia was induced by isoflurane inhalation (3% mixed with air). Subsequently mice were maintained on 1.5% isoflurane during the experiment. Once the mice were anesthetized, a small incision was made on the mid-abdomen to expose the cecum. Mice where then placed in left lateral decubitus position with appropriate support. The ambient air was kept at room temperature. The moisture of the cecum was maintained by placing moist toilettes next to the cecum. Temperature of the cecal tissue was measured at the end of each experiment and was approximately 32°C. Both lasers were focused on the same spot on the cecum surface, avoiding obvious large surface blood vessels. Oxygen measurements were obtained from the lumen (Oxyphor Micro) and tissue (Oxyphor PtR4) simultaneously at 4 s intervals. Data presented here represent an average of 5 readings taken every 12 sec over the course of 1 min. During hyper-oxygenation experiments, air was replaced with 100% oxygen in the gas mixture delivered to the mouse via a nose cone. The mice were euthanized upon completion of the experiments.

### Chemostat studies

The reactor was inoculated using a baseline fecal sample from a healthy volunteer enrolled in a dietary intervention study.^30^ Every 8 h, the volume of the cultivar was reduced to 800 mL and 200 mL of a 70:30 defined medium:pancreatic juice mixture was added. Following inoculation, the community was maintained for 14 days under anaerobic conditions. During anaerobic culturing sparging was performed using 100% N_2_, supplemented with CO_2_ as needed to maintain pH. On day 15, the small intestine cultivar was switched to oxygenated conditions and maintained for an additional 14 days. In oxygenated conditions, sparging was performed using 5% O_2_ (40 Torr) and 95% N_2_, yet the dissolved oxygen probe consistently read 0% in the cultivar indicating that the community remained in an anaerobic state despite the diffusion of oxygen. The N_2_ gas was supplemented with CO_2_ to maintain pH. Samples were collected daily in the afternoon, at the end of the feeding cycle (approximately 7.5 hrs after feeding).

### Shallow shotgun sequencing

Fecal and cecal samples from mice, as well as samples from the chemostat (days 11, 13, 14, 25, 27, 28), were sequenced using previously described methods.^44^

### Targeted metabolomics

Short chain fatty acids were quantified as previously described.^8,45,46^

### Glycoside hydrolase categorization

Glycoside hydrolases (GH) identified were individually searched within primary literature for their enzymatic roles (e.g., cleavage of plant cell wall).^47–60^ They were then assigned into four categories – plant, animal, simple sugar, or miscellaneous. GHs that have roles in more than one process were assigned to multiple categories. GHs involved in microbial mucin degradation^17^ were annotated based on their ability to degrade mucin glycans, specifically sialidases (GH33), fucosidases (GH29, GH95), N-acetyl-glucosaminidases (GH84, GH85, G89, GH20), N-acetyl-galactosaminidases (GH101, GH129), galactosidases (GH2, GH35, GH42, GH98), and endo-acting O-glycanases (GH16).

### Statistical analyses

Statistical analyses of cecum+colon length, oxygen levels, and targeted metabolomics were performed in Prism 9 (GraphPad Software, San Diego, CA) using two-tailed unpaired and paired Student’s t-tests with two-stage step up corrections for false discovery rate or Bonferroni’s corrections for multiple comparisons.^61^ Differentially abundant taxa were determined using linear mixed-effects modeling.

## Supplementary Material

Supplemental Material

## Data Availability

Shallow shotgun sequencing data is available via the Sequence Read Archive (SRA) under submission SUB13752278 and BioProject PRJNA1009084.
