# Supplementary material for: Disruption of intestinal oxygen balance in acute colitis alters the gut microbiome
Source: Gut Microbes. 2024 Jul 3;16(1):2361493. doi: 10.1080/19490976.2024.2361493 (PMC11225921; doi:10.1080/19490976.2024.2361493)
Supplement: Supplemental Material [file KGMI_A_2361493_SM6597.zip › SupplementalMethods_13May2024.docx]

**Supplemental Methods:**

*Animal experiments:* At the time of sacrifice, mice were already anesthetized under 1.5% isoflurane for the oxygen experiments and subsequently euthanized by cervical dislocation. The cecum and colon were carefully removed, and its length measured. Colonic and cecal samples were prepared for histopathology or snap frozen in liquid nitrogen and stored at -80^o^C for subsequent studies. Cecal contents were collected. Stool pellets were collected on the day of the experiment.

*Shallow shotgun sequencing:* In brief, genomic DNA was extracted using the MO BIO PowerSoil htp kit and sequencing libraries were prepared with the Nextera XT DNA Library Preparation Kit (Illumina, San Diego, CA). Libraries were sequenced on Illumina HiSeq 2500 using the paired-end 125 bp sequencing protocol. Sequence reads were processed to remove adapter sequences and low-quality reads with Trimmomatic v. 0.33^1^. Taxonomic assignments were generated with MetaPhlan2 Based on the distribution alignment length and percent identity, we removed any alignments less than 100bp in length, or having less than 95% sequence identity over the aligned region.

*Targeted metabolomics:* Short chain fatty acids were quantified in filter sterilized culture supernatants as previously described using a Waters Acquity uPLC System with a HSS T3 1.8 μm 2.1x150 mm column and a Photodiode Detector Array^2^.

*Chemostat studies:* Bioflow 320 cultivars were purchased from Eppendorf (Hamburg, Germany) and assembled according to the manufacturer’s specifications. Defined medium was Adult M-SHIME^®^ growth medium with starch purchased from ProDigest (Ghent, Belgium)^3^. Pancreatic juice was made fresh every 2-3 days^4^. For inoculation, the fecal sample was suspended at 10% wt/v in phosphate buffer within an anaerobic chamber. At time of inoculation, 10 mL of fluid was removed from the cultivar and 10 mL of the resuspended inoculums added. The cultivars were grown overnight, 16h, with temperature, pH, agitation and gas flow. Following overnight growth, the cultivars were maintained with daily feeding cycles in which the cultivar was provided fresh medium and pancreatic juice. During the experiment, all cultivars were maintained at a temperature of 37°C with agitation set to 100 rpm. The pH was kept at 7.4 ± 0.1, using 1 M NaOH and CO_2_ gas. There was a constant sparging of gas at a rate of 1L/min, with the ratio of N_2_:O_2_:CO_2_ dependent on the experimental phase. The initial volume of the cultivars was 1 L of a 70:30 ratio of defined medium: pancreatic juice. The M-SHIME^®^ growth medium contains a range of simple and complex carbohydrates, including arabinogalactan (1.2 g/L), pectin (2 g/L), xylan (0.5 g/L), glucose (0.4 g/L), mucin (2 g/L), and starch (4 g/L), as well as yeast extract (3 g/L) and peptone (1 g/L) which provide nitrogen and trace nutrients. Pancreatic juice contained 12.5 g NaHCO_3_ (Sigma-Aldrich, St. Louis, MO), 6g oxgall bile (Becton-Dickinson, Franklin Lakes,NJ) and 0.9 g pancreatin (Sigma-Aldrich, St. Louis, MO). The cultivars were connected using silicon tubing (Cole Parmer, Vernon Hills, Il) to a supply of defined medium and pancreatic juice to provide nutrition and biliary-pancreatic enzymes and to a bard urinary drainage bag (Becton, Dickinson and Company, Franklin Lakes, NJ) to collect waste. Phosphate buffer consisted of 0.8 g K_2_HPO_4_, 6.8 g KH_2_PO_4_, 0.1 g sodium thioglycolate, 15 mg sodium thionite per liter with pH adjusted to 7.

Every 8 h, the volume of the cultivar was reduced to 800 mL and 200 mL of a 70:30 defined medium:pancreatic juice mixture was added. Following inoculation, the community was maintained for 14 days under anaerobic conditions. During anaerobic culturing sparging was performed using 100% N_2_, supplemented with CO_2_ as needed to maintain pH. On day 15, the small intestine cultivar was switched to oxygenated conditions and maintained for an additional 14 days. In oxygenated conditions, sparging was performed using 5% O_2_ (40 Torr) and 95% N_2_, yet the dissolved oxygen probe consistently read 0% in the cultivar indicating that the community remained in an anaerobic state despite the diffusion of oxygen. The N_2_ gas was supplemented with CO_2_ to maintain pH. Samples were collected daily in the afternoon, at the end of the feeding cycle (approximately 7.5 hrs after feeding).

1. Bolger AM, Lohse M, Usadel B. Trimmomatic: a flexible trimmer for Illumina sequence data. Bioinformatics 2014;30:2114-20.

2. Ramsteijn AS, Jašarević E, Houwing DJ, et al. Antidepressant treatment with fluoxetine during pregnancy and lactation modulates the gut microbiome and metabolome in a rat model relevant to depression. Gut Microbes 2020:1-19.

3. Liu L, Firrman J, Tanes C, et al. Establishing a mucosal gut microbial community in vitro using an artificial simulator. PLOS ONE 2018;13:e0197692.

4. Firrman J, Liu L, Tanes C, et al. Metabolic Analysis of Regionally Distinct Gut Microbial Communities Using an In Vitro Platform. J Agric Food Chem 2020;68:13056-13067.
