# Supplementary figures and images for: Disruption of intestinal oxygen balance in acute colitis alters the gut microbiome
Source: Gut Microbes. 2024 Jul 3;16(1):2361493. doi: 10.1080/19490976.2024.2361493 (PMC11225921; doi:10.1080/19490976.2024.2361493)

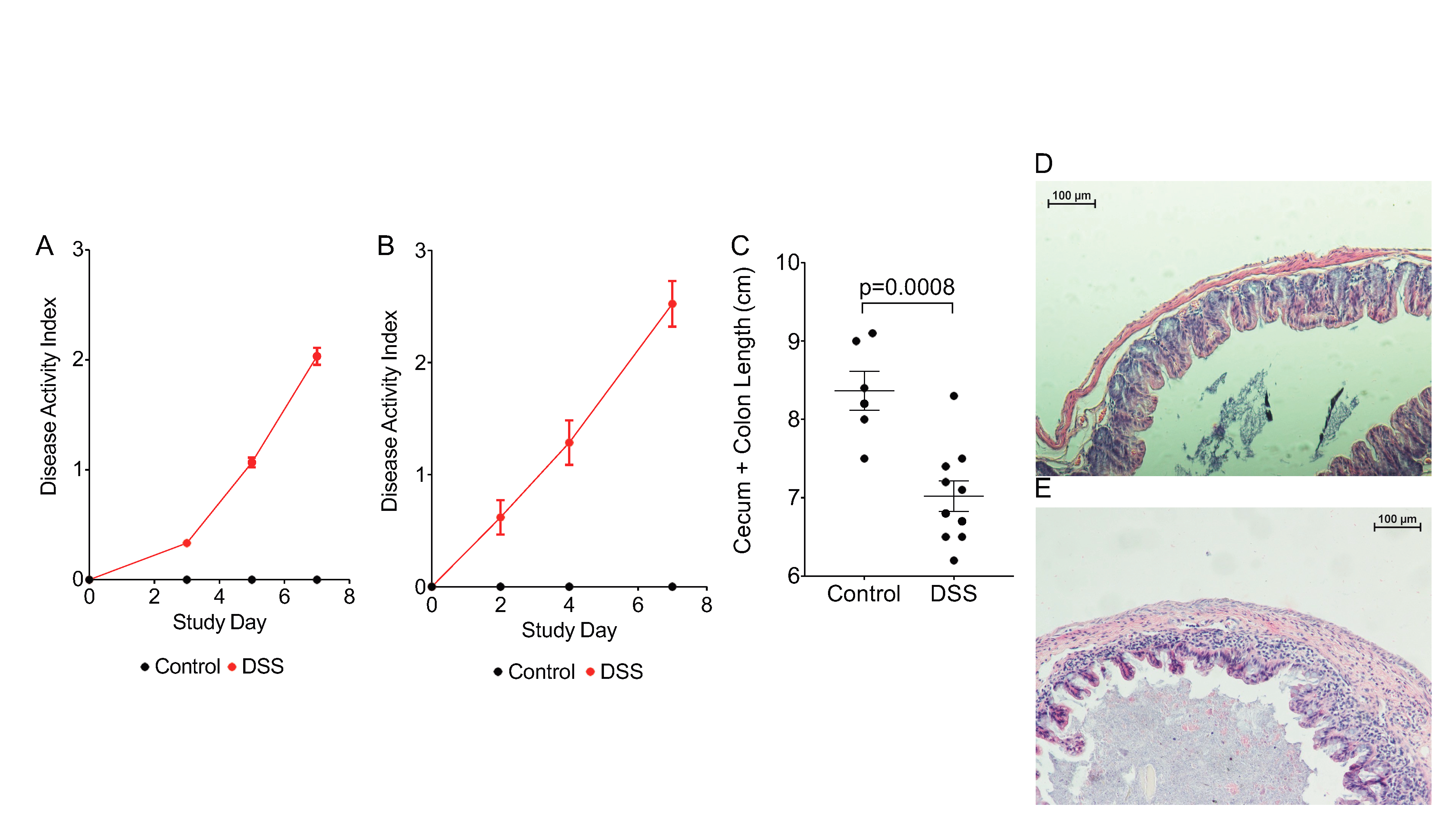

Supplement: Supplemental Material [file KGMI_A_2361493_SM6597.zip › FigureS1.tiff]

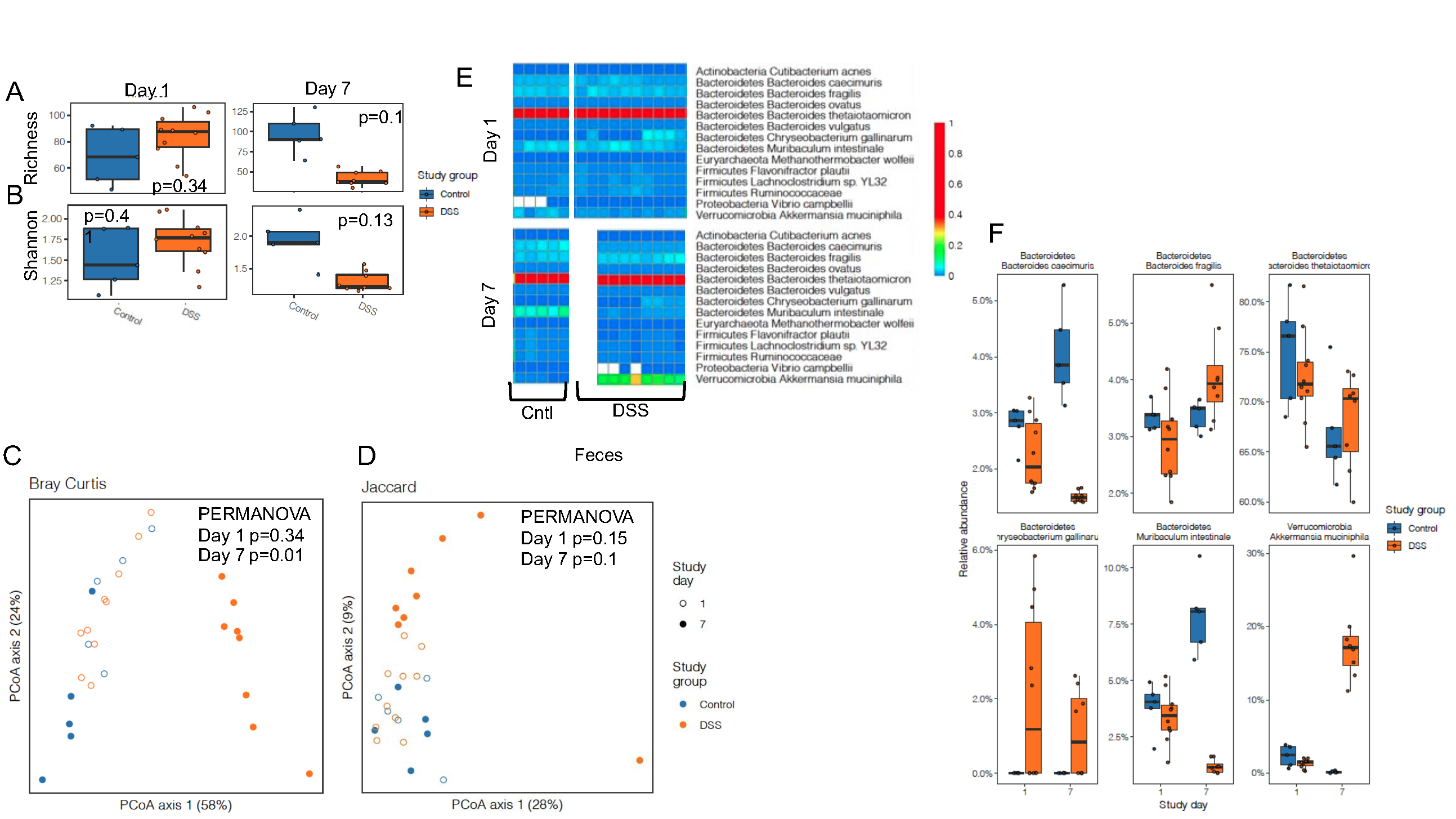

Supplement: Supplemental Material [file KGMI_A_2361493_SM6597.zip › FigureS2.tiff]
